# PReS-FINAL-2108: Long-term outcome of 114 adult JIA patients in a non-pediatric rheumatology institute in Japan

**DOI:** 10.1186/1546-0096-11-S2-P120

**Published:** 2013-12-05

**Authors:** T Miyamae, E Tanaka, T Kishi, T Matsuyama, T Igarashi, S Fujikawa, A Taniguchi, S Momohara, S Nagata, H Yamanaka

**Affiliations:** 1 Institute of Rheumatology, Tokyo Women's Medical University, Tokyo, Japan; 2 Department of Pediatrics, Tokyo Women's Medical University, Tokyo, Japan; 3 Department of Pediatrics, Nippon Medical School, Tokyo, Japan

## Introduction

The transition of adult patients with childhood-onset rheumatic disorders from pediatric to non-pediatric healthcare systems has received attention in Japan. However, the clinical course of patients transferred to non-pediatric rheumatologists has not been adequately communicated to pediatric rheumatologists.

## Objectives

To evaluate the long-term outcome of patients with juvenile idiopathic arthritis (JIA) using data from a large cohort database, IORRA (Institute of Rheumatology, Rheumatoid Arthritis), managed by Tokyo Women's Medical University in Japan.

## Methods

Of 182 patients identified from the IORRA database from 2000-2013, 114 were verified as having JIA based on the ILAR classification criteria. The transition of medical care, disease activity and health-related quality of life at the latest examination, and the contributions of biological disease-modifying antirheumatic drugs (DMARDs) were evaluated retrospectively.

## Results

The mean age of the 114 patients at the latest examination was 36.6 ± 13.3 years; there were 21 males and 93 females (81.6%). The mean age at disease onset was 11.6 ± 3.4 years, and disease duration was 25.0 ± 13.3 years. Of the 114 individuals, 106 (93.0%) had poly- or oligoarthritis; the others had systemic JIA (sJIA). Forty-five of 105 JIA patients (43%) visited non-pediatric rheumatologists from disease onset, and only one-fourth were transferred from general pediatricians or pediatric rheumatologists at a median age of 20 years. Interestingly, 26 of 105 (25%) reached transient remission in adolescence. Polyarticular JIA patients with negative rheumatoid factor (RF) showed a higher probability (41.7%) of obtaining a transient remission compared with RF-positive polyarticular JIA patients (17.8%). Disease activity assessed with DAS28 was significantly lower when disease onset was more recent (3.9 ± 1.3 for onset in the 1960s vs. 2.2 ± 1.1 for onset in the 2000s, p = 0.04), with similar results shown on the SDAI, and the CDAI. The Japanese version of the Health Assessment Questionnaire (J-HAQ) also showed improvement for those with more recent onset (1.8 ± 1.1 for onset in the 1960s vs. 0.2 ± 0.4 for onset in the 2000s, p<0.01). The induction ratio of biological DMARDs has increased for patients with more recent disease onset, with a shorter period from disease onset to induction (16.7% in the 1970s, with 27.3 ± 2.1 years to induction vs. 80.0% in the 2000s, with 5.6 ± 2.3 years to induction). Additionally, the percentage of patients requiring orthopedic surgery has decreased (53.8% before the 1970s vs. 10.0% in the 2000s). Two deaths, with causality attributed to the primary disease, occurred in sJIA patients who died from renal and/or cardiac failure due to amyloidosis at the ages of 27 and 38.

## Conclusion

From the viewpoint of pediatric rheumatologists, there are few opportunities to follow children beyond adolescence. The importance of transitioning care to non-pediatric rheumatologists with sufficient medical information is confirmed by the existence of a population with transient remission in adolescence and changes in their prognosis, with progress in rheumatology represented by biological DMARDs.

## Disclosure of interest

None declared.

